# Emerging uses of 5-aminolevulinic-acid-induced protoporphyrin IX in medicine: a review of multifaceted, ubiquitous, molecular diagnostic, therapeutic, and theranostic opportunities

**DOI:** 10.1117/1.JBO.30.S3.S34112

**Published:** 2025-10-08

**Authors:** Brian W. Pogue, Bin Chen, Marien I. Ochoa, Arthur Petusseau, Aiping Liu, Angela L. F. Gibson, Edward V. Maytin, Brian C. Wilson

**Affiliations:** a Thayer School of Engineering at Dartmouth, Hanover, New Hampshire, United States; b University of Wisconsin School of Medicine and Public Health, Department of Medical Physics, Madison, Wisconsin, United States; c St Joseph’s University, Department of Pharmaceutical Science, Philadelphia, Pennsylvania, United States; d University of Wisconsin School of Medicine and Public Health, Department of Surgery, Madison, Wisconsin, United States; e Cleveland Clinic, Lerner Research Institute, Department of Biomedical Engineering, Cleveland, Ohio, United States; f Princess Margaret Cancer Centre/University Health Network and University of Toronto, Toronto, Ontario, Canada

**Keywords:** fluorescence, photodynamic, contrast agent, imaging, therapy

## Abstract

**Significance:**

5-Aminolevulinic acid (5-ALA) is a medical pro-drug used to induce the intracellular production of protoporphyrin IX (PpIX) via the heme synthesis pathway. Discoveries in mechanisms and developments in novel applications still continue with this uniquely endogenous intracellular optical system.

**Aim:**

Understanding and exploiting the growing uses can be advanced through a survey of knowledge on the mechanisms and biokinetics of 5-ALA administration, partitioning, PpIX production, localization changes, clearance mechanisms, biological interactions, and methods for unique activation methods in both diagnostic and therapeutic applications.

**Approach:**

The current medical uses of PpIX are reviewed, separating into therapeutic and diagnostic areas, and the expansion and lateral growth areas are outlined.

**Results:**

Initially approved for photodynamic therapy of skin lesions, fluorescence diagnostic indications later developed to guide surgical resection in bladder cancer and glioma. Today, the 5-ALA-PpIX system’s spatial-temporal complexity in photophysics and pharmacokinetics continues to lead to more uses, such as photodynamic priming to alter tissue, fast intracellular tissue oxygen sensing, infection, and burn imaging and therapy.

**Conclusions:**

The 5-ALA-PpIX system has broad potential partly because of the ubiquity of the heme synthesis across many cell/tissue types, combined with natural selectivity, unique pharmacokinetics, bright fluorescence, and sufficiently strong singlet oxygen production.

## 1 Introduction

**Fig. 1:**
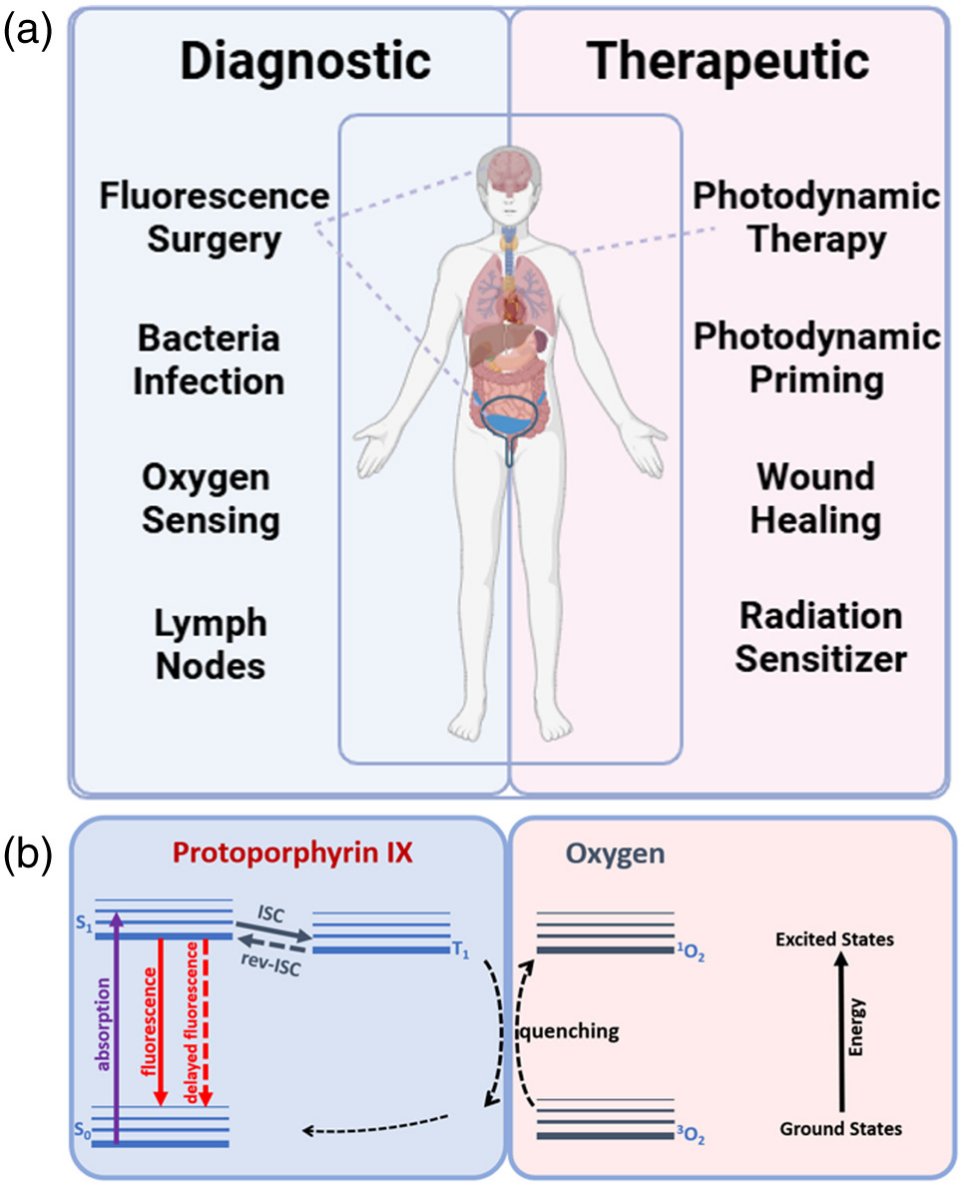
PpIX uses are shown (a), for diagnostics and therapeutics, ranging from clinically established applications (upper) to investigational applications (lower). In panel (b), the Jablonski energy-level diagram for PpIX shows the fluorescence emission (diagnostic uses) and the oxygen-quenching process used for most therapeutic applications.

Endogenous protoporphyrin IX (PpIX) has been used in multiple applications in medicine, most notably in clinical photodynamic therapy (PDT) and fluorescence-guided surgery (FGS),^1^^–^^5^ and has been widely studied as the cause of a group of light-sensitizing disorders termed porphyria. However, it has also seen expanded investigative use in other applications where this porphyrin is naturally present or can be induced, such as in bacteria^6^^–^^10^ and areas of inflammation or damaged tissues. It also has unique signatures that are being explored in measuring tissue hypoxia and lymphatic flow and in therapeutic applications such as neoadjuvant priming or stimulation of tissue healing. Many of these areas are highlighted in Fig. 1(a). This survey examines the unique features of this PpIX and how its ubiquitous metabolic production, combined with its whole body biodistribution and kinetics, make it relevant for therapy and/or diagnostics in a range of body sites and conditions. The two key applications stem from the fact that PpIX produces fluorescent light for diagnostics as well as singlet oxygen for therapy.

PpIX is naturally present throughout the body, but its production can be specifically stimulated, either systemically or locally, through the administration of exogenous 5-aminolevulinic acid (5-ALA), which is a rate-limiting precursor in heme biosynthesis. Many of the applications are enabled by localization or partitioning of the resultant PpIX in specific tissues or biofluids. In addition, temporal changes occur in localization or effect, as both 5-ALA and PpIX have intracellular and extracellular phases, leading to either local or systemic distributions, and organ/tissue-specific clearance kinetics. It is worth noting that 5-ALA can sometimes be abbreviated as just ALA or δ-ALA in the literature, where we use the most conventional 5-ALA term to denote the molecule.

Widespread clinical adoption of 5-ALA-induced PpIX as a PDT agent came in the early 1990s with the demonstration that the topical application of a 5-ALA solution onto the skin led to robust production of PpIX and that this was a potent photosensitizer for lesions such as actinic keratoses (AKs).^5^^,^^11^^–^^13^ This unique approach of endogenous synthesis of PpIX from exogenous 5-ALA allows for effective PDT treatment of many lesions, not just in the skin but in several squamous tissues, bacteria, and even solid tumors. In the United States, its use is currently restricted to AK treatment, but it is in routine use in several other countries for superficial basal cell carcinoma. The 5-ALA administration routes approved for human use now include topical, intravesical, and oral (systemic),^14^^–^^17^ with the strong tumor-specific fluorescence signal of PpIX being used to guide surgical resection in, for example, bladder cancer and glioma. The use in bladder cancer resection has been adopted for several decades,^18^^–^^22^ although its acceptance in urologic surgery has been reserved for more complex cases.^23^ Its use in glioma resection has been approved for nearly two decades in Europe and a decade in the United States,^24^^–^^26^ with clinical adoption growing steadily.^27^ Individual studies have examined its role in other surgeries, but few other approvals have occurred to date. Part of the challenge is the ubiquitous nature of PpIX synthesis that can reduce tumor-to-normal tissue contrast in some organs. However, due to the low activity of enzyme ferrochelatase, a lot of tumors have a high amount of endogenous PpIX, even without any external ALA supply, which in principle can lead to naturally occurring PpIX for diagnostic measurement itself, but can be at lower levels, yielding limited sensitivity. It is notable that most successful applications have an inherently low normal tissue background production of PpIX.^28^ PpIX is produced within mitochondria but then diffuses through tissue and then through the bloodstream within hours, reducing the contrast over time.^29^^–^^31^ The key feature that seems to allow selective use in skin lesions, glioma, and bladder tumors is intralesional localization,^32^ with minimal PpIX in the surrounding normal tissue, although ongoing investigators are examining if this is due to 5-ALA penetrance and/or to metabolic differences between tumor and normal tissue.^33^^,^^34^ Current established and investigational uses of PpIX are illustrated in Fig. 1(a).

The large Soret absorption peak around 400 nm has been used mainly for fluorescence imaging (blue light), whereas most PDT treatments use the longest Q-band around 635 nm (red light) to obtain maximal effective tissue depth. The PpIX energy level kinetics and transitions for fluorescence, delayed fluorescence, and oxygen quenching are outlined in Fig. 1(b). There is a high efficiency for intersystem crossing (ISC) to the triplet state, which has an energy spacing resonant with ground-state oxygen to yield efficient (quantum-mechanically permitted) collisional energy transfer to triplet ground-state oxygen, often termed triplet quenching. This produces excited singlet-state oxygen, {^1O2}, which is the primary active species that induces cellular toxicity in PDT. Importantly, the fluorescence and singlet oxygen pathways are competitive so that each molecule can only generate either one quantum event. There is no US approval to date of clinical applications where 5-ALA-PpIX is used as both a diagnostic and a therapeutic, although many clinical trials have investigated this^35^ and it is routinely used this way in Canada and Brazil.^36^^–^^38^ Beyond this, there are additional unique photophysical properties, including reverse intersystem crossing [rev-ISC in Fig. 1(b)] from the triplet to the singlet state, producing oxygen-dependent delayed fluorescence.^39^^,^^40^ This has been adopted clinically in Europe for tissue oxygen monitoring and is also being explored for imaging of hypoxia *in vivo*.^41^^–^^43^ In addition, the production of PpIX in areas of wounds, burns, and bacterial infection is of emerging diagnostic interest. The photophysics of how to excite PpIX have been extensively investigated, with tradeoffs between the use of blue versus red light, with the latter allowing deeper excitation for sensing or therapy, whereas the blue provides more robust excitation, albeit in superficial tissues. Moreover, multiple Q-bands exist through the blue-green wavelengths, allowing for excitation of PpIX across much of the visible spectrum of light.

There are unique aspects of the PpIX-mediated photobiology that enable possible therapeutic effects beyond standard PDT. A first example is low-dose photosensitization or photodynamic priming (PDP)^44^^–^^46^ that can induce a cascade of intra- and extra-cellular signaling, leading to adaptive immune responses as a potential neoadjuvant therapy. Second, a dominant factor seen in low-dose PDT has been vascular dilation or occlusion^47^ and a small PDT dose given hours before the main treatment can result in large improvements in response.^48^^–^^53^ In addition, PpIX at low concentrations in normal tissues has been postulated as a possible source of photobiostimulation.^44^^–^^46^ Finally, treatment of other tissue damage such as burns has been examined and appears promising as a stimulatory adjuvant therapy.^54^ Each of these approaches is discussed further below.

**Fig. 2:**
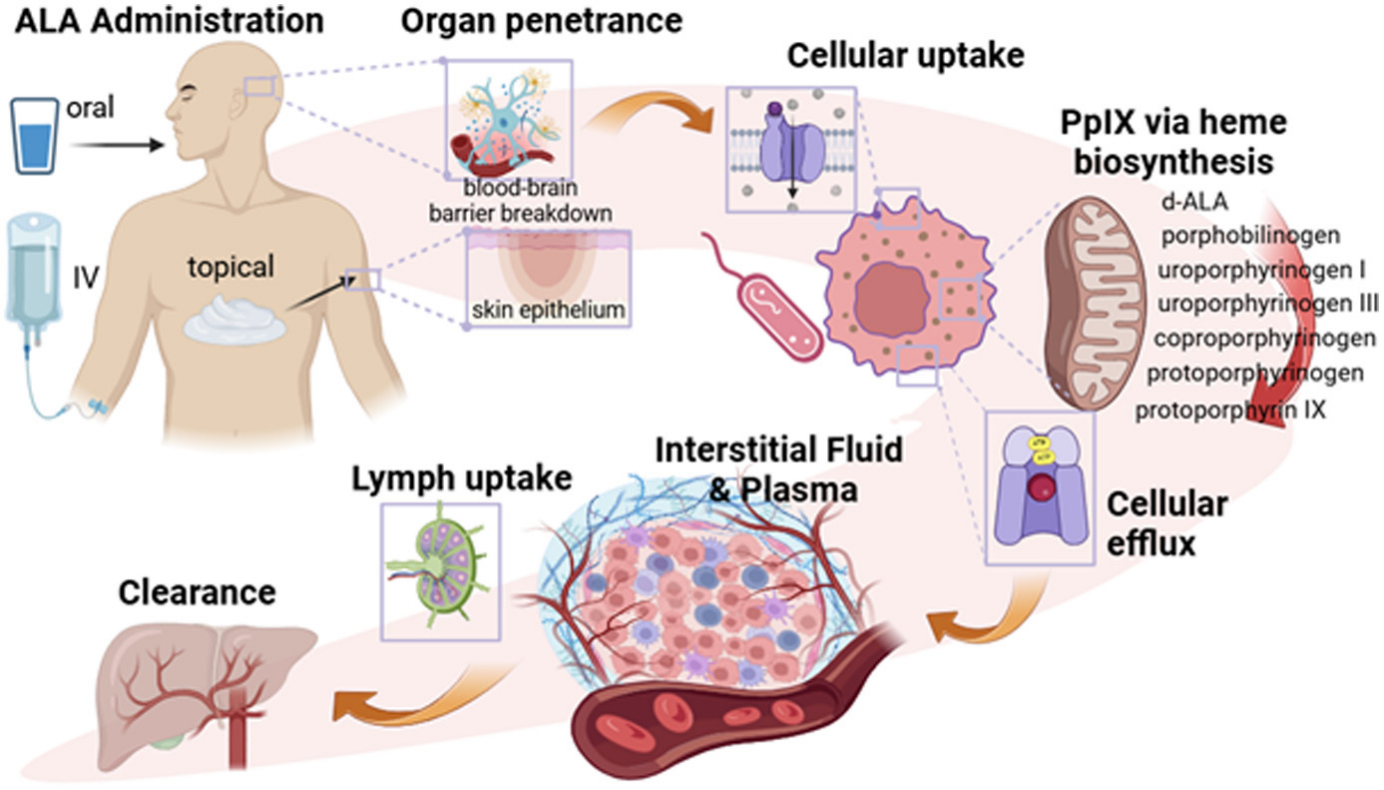
Pharmacokinetic pathway from 5-ALA to PpIX is illustrated, starting with administration and penetrance of 5-ALA, through to cellular uptake and intracellular PpIX synthesis. Excess intracellular PpIX is pumped out of cells and has diffusion/convection transport throughout interstitial fluid spaces and is drained via plasma and lymph fluids, ultimately being processed by the liver and cleared through the GI tract.

In summary, some of the key factors to the multitude of real and potential clinical applications of 5-ALA-PpIX in medicine are the following, as illustrated in Fig. 2:

1. Ubiquitous heme metabolism pathway that utilizes 5-ALA in PpIX synthesis in many prokaryotic and eukaryotic cells, producing a signal that comes from within defined areas of cells and tissue.
2. High biocompatibility and tolerance of 5-ALA in different administration routes, followed by well-tolerated and efficient clearance of PpIX within 24 h.
3. Bright fluorescence at red-near infrared (NIR) wavelengths.
4. Multiple excitation wavelengths from blue to red.
5. Naturally low 5-ALA uptake or PpIX conversion in some normal tissues (e.g., skin, brain, bladder), providing natural selectivity in some malignancies.
6. Cellular efflux and relocation of PpIX outside producing cells over several hours, providing temporal windows of contrast based upon enhanced permeability and retention effect (EPR).
7. Extracellular diffusion-convection driven flow into lymph, plasma, and processed by the biliary system for colon clearance.
8. Naturally high yield of PpIX quenching by oxygen to efficiently produce singlet oxygen for photosensitization or oxygen-dependent sensing, and cycling to generate this repeatedly until photobleaching occurs.
9. Sub-threshold cellular and vascular effects seen during initial photosensitization, which can be modulated to be stimulatory, apoptotic, or necrotic in nature, depending upon the light dose.

## 2 5-ALA, Derivatives, Formulations, and Trade Names

Medical use of 5-ALA-PpIX has been facilitated in several ways, including alteration of 5-ALA using methyl or hexyl esters to enhance lipophilicity, or customized administration using formulations to optimize tissue penetrance. There have also been multiple administration methods investigated, given the ubiquitous and biocompatible nature of the small 5-ALA molecule (MW: 131 Daltons).^55^^,^^56^ Dermatological applications benefitted from extensive research into topical formulations that improve tissue penetrance and specificity, with Levulan™ being the longest-used 5-ALA variant, applied as an aqueous solution with alcohol. In Europe, Metvix™, a methyl-ALA molecule that has superior penetrance, is a cream formulation. A nanoemulsion formulation, Ameluz™, delivered as a gel, is approved in both Europe and the USA.^57^ Extensive studies have examined versions of microneedle penetration,^58^^–^^60^ as well as the use of electric pulse electroporation,^61^ heat,^62^^,^^63^ occlusion,^64^ fractional laser treatments,^65^ and adjuvant application of iron chelators,^66^^,^^67^ with each showing some level of increased PpIX production. A reduced drug-light time interval has become common, as short as 30 min, to reduce pain during the light delivery in PDT treatments of skin lesions.

For surgical guidance, the first widely adopted application was in the bladder, where topical instillation was developed using an aqueous lipophilic solution of ester hexyl-ALA (Hexvix, marketed as Cysview™).^68^^–^^73^ This optimizes penetrance across dysplastic bladder urothelium and provides good fluorescence contrast in lesions for surgical guidance. An international prospective phase III clinical trial demonstrated that tumor resection under the guidance of hexyl-5-ALA fluorescence increased the median time to tumor recurrence to 16.4 months compared with 9.4 months in patients treated with the standard white light surgery (P=0.04). This led to the FDA approval of hexyl-5-ALA for detecting superficial bladder cancer in 2010.^74^ However, a more recent randomized clinical trial in the United Kingdom showed similar rates of tumor recurrence at 3 years for both groups,^75^ and it is unclear why the short-term benefits from fluorescence-guided tumor resection shown in the earlier study^74^ did not lead to a long-term tumor control.

Oral administration of 5-ALA is used for fluorescence visualization of glioma during neurosurgery,^76^^,^^77^ primarily using the 5-ALA hydrochloride formulation called Gleolan™ in North America and Gliolan™ in Europe.^27^^,^^78^^,^^79^ Deep-tissue imaging experimental situations that employ PpIX fluorescence often require systemic 5-ALA administration, although they could utilize rinse formulations, inhalation, intravascular injection, or direct injection,^15^^,^^80^^,^^81^ including endoscopic reachable surfaces, biopsy needle introduction, and experimental optical imaging techniques. This has been used for clinical trials in various organs, including for Barrett’s esophagus^82^^–^^84^ and solid tumors such as nodular basal cell carcinoma and others.^85^^,^^86^ Inhaled administration has also been demonstrated in pilot trials for bronchial PDT.^87^ Measurement of PpIX fluorescence in a target tissue is often used to assess PDT potential, the selectivity is assessed as the ratio of signal in malignant versus benign/normal tissue,^88^^,^^89^ and online spectral monitoring has been developed for glioma.

## 3 Protoporphyrin IX Production and Clearance

**Fig. 3:**
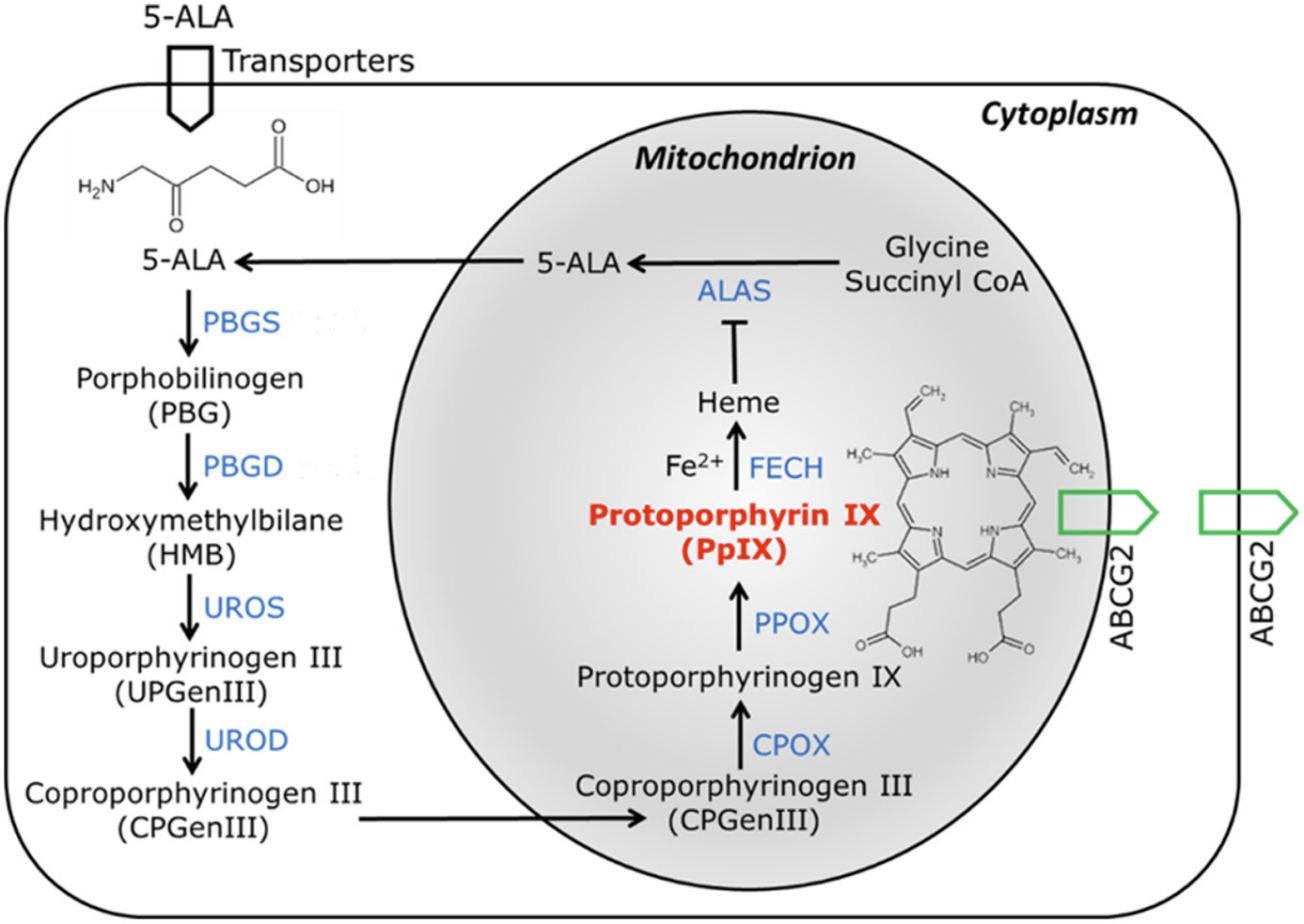
5-ALA or derivatives are administered topically or orally and are metabolized in the endogenous cellular heme-biosynthesis pathway to produce PpIX. This pathway involves eight enzymes in sequence: 5-ALA synthase (ALAS, the rate-limiting enzyme for exogenous 5-ALA); porphobilinogen synthase (PBGS); porphobilinogen deaminase (PBGD); uroporphyrinogen III synthase (UROS); uroporphyrinogen III decarboxylase (UROD); coproporphyrinogen III oxidase (CPOX); protoporphyrinogen IX oxidase (PPOX); and ferrochelatase (FECH). Exogenous 5-ALA bypasses the rate-limiting step of ALAS, resulting in intracellular PpIX accumulation, typically due to insufficient FECH or ${\mathrm{Fe}}^{2+}$ to synthesize heme. The ABCG2 transporter localized in mitochondrial and plasma membranes reduces intracellular PpIX concentration by transporting PpIX out of the cell.

Production of PpIX is part of the heme biosynthesis pathway present in all nucleated cells.^90^ This comprises eight consecutive steps catalyzed by eight different enzymes localized in the mitochondria or cytosol (Fig. 3). In addition to the spatial compartmentation of these enzymes, the pathway is regulated by a feedback inhibition mechanism whereby accumulation of the heme end product inhibits the first enzyme of the pathway, 5-ALA synthase (ALAS), and temporarily decreases the pathway flux. However, when 5-ALA is administered exogenously by either topical application or systemically, it enters cells through the peptide transporters on the cell membrane^91^ and bypasses this rate-limiting step. This in turn results in intracellular accumulation of intermediates, including uroporphyrinogens, coproporphyrinogens, and PpIX. Although a fraction of uroporphyrinogens and coproporphyrinogens can be oxidized to produce uroporphyrins and coproporphyrins with fluorescence and photosensitizing activity, PpIX is the predominant fluorescent porphyrin produced in both tumor and normal cells after 5-ALA administration.^92^ In addition, PpIX exhibits much stronger photosensitizing activity than coproporphyrins and uroporphyrins,^93^ and there are spectral shifts between these porphyrin types allowing for some level of differentiation. Thus, the diagnostic and therapeutic uses of 5-ALA-PpIX for tumor fluorescence imaging and PDT rely on the preferential PpIX accumulation in tumors over the surrounding normal cells.

Intracellular PpIX levels in both tumor and surrounding normal cells change dynamically following 5-ALA administration. Tumor fluorescence imaging and PDT should be performed at drug-light intervals (DLIs) when PpIX in the tumor is both sufficient for fluorescence detection or effective treatment and selective to ensure high contrast and a good therapeutic window. The time-dependent intracellular PpIX concentration depends on several factors, including the accessibility to 5-ALA and its cellular uptake, 5-ALA-PpIX biosynthesis, PpIX bioconversion, and PpIX efflux and passive diffusion, and these have been extensively reviewed in previous papers and reviews.^1^^,^^3^^,^^34^^,^^94^^–^^99^ These determinants have been explored for optimal tumor-cell specific imaging and targeting.

The difference between tumor and normal tissues in the physiological barrier to exogenously applied 5-ALA has been exploited to achieve high tumor selectivity with ointment in skin and intravesical rinsing in the bladder. This is most apparent in topical administration to visualize/treat superficial skin and bladder lesions. The outermost keratin layer in skin and the innermost apical layer of the bladder urothelium serve as barriers to 5-ALA so that disruption by underlying tumor growth facilitates greater access to locally applied 5-ALA than in the surrounding normal tissue. PpIX fluorescence in skin cancer can be seen almost immediately after the topical application of 5-ALA.^100^ Changing from topical to systemic administration will result in PpIX production in many normal tissues and, over several hours, can generally reduce tumor selectivity from increased PpIX levels in the interstitial fluid, lymph, and plasma.^13^^,^^101^^,^^102^ However, compromised blood-brain barrier (BBB) in (high-grade) gliomas gives tumor cells ready access to circulating 5-ALA compared with normal brain cells,^103^ particularly in white matter where the majority of adult gliomas arise. This difference between high-grade gliomas and normal brain tissues enables PpIX fluorescence-guided tumor resection.^104^ Most low-grade gliomas display significantly lower positive 5-ALA-PpIX fluorescence,^105^^,^^106^ which is a limiting factor with current imaging systems^107^ but has been quantified well with fiberoptic probe devices.^26^^,^^108^ There appears to be a sufficient PpIX present for further resection guidance if the instrumentation has sufficient detection sensitivity, such as in a contact probe.

Molecular mechanisms underlying preferential PpIX tumor accumulation have been extensively investigated, although knowledge is still incomplete. As discussed above, enhanced 5-ALA-PpIX production in tumor cells is associated with alterations in heme biosynthesis enzymes, mitochondrial function, and porphyrin transporters.^3^ Some of those changes have been shown to occur after oncogene transformation,^109^^–^^111^ suggesting that enhanced 5-ALA-induced PpIX accumulation in tumor cells occurs as part of the resulting overall metabolic changes. In squamous cancers, major changes in the relative availability of enzymes that regulate PpIX synthesis have been well documented, especially after the introduction of agents that enhance squamous differentiation and, thereby, increase PpIX accumulation through differential regulation of coproporphyrinogen synthase and ferrochelatase.^112^^–^^115^

Intracellular PpIX is cleared by several processes, including PpIX-to-heme bioconversion catalyzed by ferrochelatase and active PpIX efflux by the ABCG2 transporter. Inhibition of PpIX clearance has been demonstrated to increase intracellular PpIX levels.^1^ Due to the lack of pharmaceutical ferrochelatase inhibitors, iron chelators have been used to inhibit PpIX-to-heme bioconversion by chelating with the labile ferrous iron (${\mathrm{Fe}}^{2+}$) required for the ferrochelatase-catalyzed reactions. Thus, with less ${\mathrm{Fe}}^{2+}$ available to react with PpIX to form heme, clinical iron chelators such as deferoxamine and deferasirox increase intracellular PpIX accumulation. However, deferoxamine has been shown to be less effective in tumor cell lines with low ferrochelatase activity^116^ and its effectiveness in increasing PpIX levels decreases with longer 5-ALA incubation times.^117^ Compared with deferoxamine, the inhibition of ABCG2-mediated PpIX efflux appears more effective for enhancing PpIX accumulation in tumor cells *in vitro*.^118^ As a transporter involved in multidrug resistance to chemotherapeutic drugs and often overexpressed in a variety of tumor tissues, ABCG2 reduces intracellular PpIX levels by actively pumping PpIX out of tumor cells.^119^ Hence, suppression of ABCG2 activity enhances tumor PpIX fluorescence and sensitizes tumor cells to 5-ALA-PpIX mediated PDT. The identification of clinically relevant ABCG2 inhibitors has enabled preclinical studies and potential clinical trials.^120^

Systemic clearance of PpIX has been well studied over decades and has shown systemic plasma accumulation hours after topical skin 5-ALA administration, with clearance via the liver and intestines.^121^^,^^122^ The systemic administration of 5-ALA produces PpIX production in many body tissues at some level, making the pharmacokinetic changes harder to study.^123^ The lowest level tissues appear to be muscle and brain.^124^ Topical application has been used in many dermatological applications, where PpIX concentration in plasma peaks at ∼4±1  h and is largely undetectable by 24 h.

## 4 Photodynamic Therapy: Current and Future Indications

Dermatological applications of PDT with 5-ALA-PpIX are the largest clinical and commercial success of PDT in oncology to date, with three major approved formulations and a range of light sources.^125^^–^^127^ Currently, regulatory approvals in the United States and Europe have been granted for an alcohol-based solution of 20% 5-ALA (Levulan™) paired with blue light; a cream with 16% methyl-5-ALA (Metvix™) paired with red light; and a nanoemulsion gel with 10% 5-ALA (Ameluz™) paired with red light. These are approved for the treatment of actinic keratoses (AK) in the United States and Europe and also in Europe for superficial basal cell carcinoma (BCC). A phase III trial for US FDA approval of Ameluz™ and red light for BCC is currently underway as of 2025. For both AK and BCC, there is considerable variability in PpIX production across lesions and between patients, and this seems to limit efficacy.^128^^,^^129^ PpIX fluorescence to guide PDT is established in many treatment centers in Brazil^130^ and extending to neighboring countries. However, the approved use in most US and European centers is to treat without any measurement of PpIX, and thus, failures or nonresponding lesions may be due to limited PpIX present. It is common to accept that the lesions may be cleared through repeated treatment.^131^^–^^134^

The timing of light delivery following 5-ALA administration for these applications has evolved, driven in part by the need to minimize the localized pain during light application. In the original FDA-approved protocols, the recommended drug-light interval was 14 to 18 h with Levulan™ before blue light or 3 h with Metvix™ or Ameluz™ before red light. In all cases, patients experience stinging pain during PDT illumination that is often so intense as to terminate the treatment session.^135^^,^^136^ Hence, the drug-light interval was shortened by practitioners, and there is currently no universal time interval for PDT of AK.^137^ However, new controlled studies have indicated that effective pain-free PDT regimens are possible. For example, a trial in 2020 examined patients with AK on the face and scalp receiving a conventional PDT regimen on one half of the face (20% 5-ALA for 1 h, followed by blue light for 16 m 40 s) and the controlateral side receiving blue light starting immediately after 5-ALA application (no pre-incubation) for either 30, 45, or 60 min.^138^ Stinging pain was reported with the conventional regimen but little or no pain with simultaneous incubation-illumination. Importantly, AK lesion clearance was comparable across the treatment regimens.^138^ Other groups have shown similar results using short-incubation PDT with blue light.^139^^–^^141^ In 2025,^142^ a study using red-light PDT piloted three short-incubation regimens that attempted to mimic the prior approach with blue light. The results showed that 10% 5-ALA (Ameluz™) with a drug-light interval of 10 min followed by 20 min of red light was essentially pain-free and that the AK lesion clearance rates were statistically noninferior to a standard (painful) regimen of 60 min of Ameluz™ followed by 10 min of red light.^142^ The most likely reason for lack of pain with the new protocol is that PpIX is photobleached soon after it is generated within target cell mitochondria, thereby preventing accumulation and diffusion into surrounding nerve endings (pain fibers) in the skin.^143^

Fractionated light delivery was investigated in a large clinical trial to treat BCC and found to be substantially better than a single light fraction in terms of lesion clearing.^144^ It is unclear what has limited wider adoption of this approach, although one factor is the longer overall procedure time. Nevertheless, the observation that using fractionated light can increase treatment efficacy through mechanisms such as vasodilation, which oxygenates tissue and improves 5-ALA delivery and PpIX production, has stimulated its use in other areas such as photodynamic priming, discussed below.

An interesting trend in many countries, especially those with public health care, is the activation of 5-ALA-PpIX using sunlight. The so-called “daylight PDT” was first introduced in Denmark^145^^,^^146^ and has been extensively studied in clinical trials,^147^ with the general consensus being that it is safe and effective.^146^^,^^148^ There are even studies examining the effects of longitude to determine if there is sufficient daylight in winter to perform these treatments.^149^ Although less successful in the winter months, there is an opportunity to use daylight PDT when delivered carefully with ∼2  h of sunlight or delivered indoors through a plate-glass window in a clinic setting where solar dosimetry measurements can guide treatment time.^150^

Beyond dermatology, 5-ALA-PDT has been investigated for many solid tumors in several body sites, and this continues to be an active research field.^151^^,^^152^ There are several interesting associated innovations, such as the concept of implicit dosimetry^153^ in which monitoring fluorescence photobleaching allows for direct quantification of treatment progression.^154^^–^^159^ 5-ALA has been extensively studied to treat bladder cancer, with at least six clinical trials in the late 1990s to early 2000s:^160^ patients with bladder carcinoma *in situ* or cancer resistant to conventional therapies were treated using blue, red, or white light after intravesical administration of 5-ALA.^161^ In addition to demonstrating the safety and feasibility of 5-ALA-PDT, favorable responses were also reported in all these trials but did not lead to governmental approval. Commercial adoption of the treatment of large solid tumors with 5-ALA-PpIX has not been obtained although several studies in glioma appear promising, as discussed below.

Around the same period, more lipophilic hexyl-ALA was developed with the goal of increasing tissue penetration by overcoming the limitation of 5-ALA’s hydrophilicity.^162^ Although hexyl-5-ALA instilled into the bladder was eventually approved for bladder cancer fluorescence detection, PDT with hexyl-ALA has not been adopted. Limited reports to date suggest low therapeutic efficacy, possibly due to rapid washout, dilution by urine, and low and heterogeneous PpIX production in tumors.^163^ These limitations have promoted the development of new prodrugs, nanoformulations, and combination strategies to enhance tumor PpIX levels. One of the limiting factors is the expression of ABCG2 that results in low and heterogeneous PpIX levels in bladder cancer cells after the administration of 5-ALA or hexyl-ALA.^164^^,^^165^ *In vitro*, the inhibition of ABCG2-mediated PpIX efflux has been shown to enhance PDT response by increasing PpIX accumulation in tumor cells.

Numerous preclinical studies have shown that 5-ALA-PDT induces brain tumor cell apoptosis/necrosis and exhibits anti-tumor effects in various tumor models.^166^ Early phase clinical trials with limited patient cohorts have demonstrated the safety and feasibility of intraoperative PDT following glioblastoma resection with 5-ALA-PpIX fluorescence, i.e., employing 5-ALA-PpIX as a theranostic agent. Following FGS, the tumor bed was irradiated with one or more laser diffusors^35^ or a balloon device filled with light-diffusing fluid.^167^ In both trials, patients were also treated with chemotherapy and radiation therapy. In the INDYGO trial where balloon illumination was used, all 10 patients received the same adjuvant therapy (60 Gy + temozolomide) after PDT. Interstitial PDT of GBM has been examined clinically in two safety studies in Europe (GL01, NOA11), with long-term follow-up and analysis of survival showing a large fraction of patients with prolonged survival.^97^^,^^168^^–^^170^ A recent update showed encouraging patient survival data, leading to an ongoing clinical study in the United States.^171^ Interstitial treatment with dosimetry guidance for the treatment has proven more effective and is widely considered safer than simply prescribed delivery of light.^152^^,^^172^ Despite being well studied and higher efficacy, there has been a resistance in commercial suppliers of PDT to making the treatments more complex.^173^ Further study of this issue is warranted, given the commensurate benefits of delivery of effective doses that have proven tumor control capability.

## 5 Fluorescence-Guided Surgery: Diagnostic Indications and Visualization Systems

Guiding tumor resection by fluorescence imaging and/or point spectroscopy is likely the most widely used 5-ALA-PpIX technique. Notably, it is one of only a few small molecules that have been successfully used to guide surgery based on tissue metabolism and by far the most successfully introduced. The success of this agent and its ease of use have far outweighed any of its biophysical or photochemical limitations. Its use in the bladder dates back to the work of Kriegmair and colleagues that led to approval in Germany in the early 1990s.^18^^,^^174^^,^^175^ Cysview™ and the Karl Strorz D-Light fluorescence cystoscopy system have been in continuous clinical use subsequently.^21^^,^^176^^,^^177^ Fluorescence imaging-enabled surgical microscopes include the Zeiss BLUE 400^®^, BLUE 400 AR^®^ and OPMI Pentero 900^®^ systems (Carl Zeiss Meditec)^178^ and the Leica FL400^®^ and M530 OHX^®^ systems (Leica Microsystems).^179^ These have been widely adopted, mainly in neurosurgical guidance for glioma. These instruments have a large field of view (FOV) (typically, ∼2 to 4 cm diameter) but have relatively low spatial resolution (∼mm) and, because of the large working distance (∼50  cm) to allow surgical access, have limited sensitivity to low PpIX fluorescence levels.^179^ Endoscopic systems with smaller FOV have been developed to provide better resolution.^180^ Scanning endoscopic systems have also been proposed for a larger FOV.^181^ Most commercial systems use broadband blue light excitation (375 to 440 nm), with the red PpIX emission captured by an RGB camera,^177^ and are packaged to allow switching between standard white-light reflectance and PpIX fluorescence imaging. The added clinical value of PpIX imaging is still being debated.^182^^–^^184^ However, their use in complex bladder surgeries and cases where scar tissue is present remains valid.^178^

The use of 5-ALA-PpIX fluorescence imaging in glioma surgery was accelerated by a key clinical trial by Stummer and colleagues in 2006 that showed increased short-term survival.^76^ Visualization of the red PpIX fluorescence was achieved with blue excitation, implemented on either the FL400 Leica or BLUE400 Zeiss Pentero neurosurgical microscopes. The latter uses a 455 nm long-pass filter after excitation with violet-blue (375 to 440 nm) light from a filtered xenon lamp.^185^^,^^186^ Quantitative measurement of PpIX in neurosurgery has been intensively examined,^187^^–^^190^ either in the point-spectroscopy mode with a fiber-optic probe incorporating both fluorescence and diffuse-reflectance spectroscopy, or with wide-field imaging also incorporating correction algorithms. In each case, the aim is to correct for the variable tissue absorption and scattering, enabling higher sensitivity (lower minimum detectable PpIX concentration) and more objective decision-making in surgical resection.

Confocal imaging of PpIX *in situ* and in tissue samples *ex vivo* has been used to improve tumor margin localization^191^^–^^193^ but is not widely adopted. It seems likely that the speed, tissue coverage, and ability to interpret the images all limit neurosurgical adoption, although research continues. Research is also ongoing into different macroscopic visualization and guidance approaches, such as wearable loupe glasses that are designed to capture fluorescence.^194^ As head-mounted loupes are commonly used in microsurgery,^195^ the ability to incorporate fluorescence imaging into these is of interest, but a key limitation has been to preserve their light weight and ease of use. It seems likely that advances in consumer technologies for virtual reality may ultimately enable clinical adoption.^195^^–^^202^

In general, endogenous (auto)fluorophores such as PpIX typically have lower quantum efficiency than optimized exogenous agents.^203^ Comparisons have been made between the use of blue and red PpIX excitation, the trade-off being between effective depth of imaging and PpIX detection sensitivity.^204^^,^^205^ Tomographic configurations have also been investigated for sub-surface PpIX fluorescence detection,^205^^,^^206^ and structured-light quantitative imaging is under development for improved depth determination.^207^ Technical challenges include the inverse problem that is computationally intensive and limits the imaging frame rate. Improving the detection sensitivity is of significant interest because it can allow detection of lower PpIX concentration in lesions that may not be visualizable by the camera systems. Fiberoptic probes have been shown to provide up to 10× improvement in the detectable minimum PpIX concentration,^26^ although at the cost of speed and sampling density. Detection based on PpIX emission lifetime has been investigated,^208^ as has spectral-based fitting.^209^ Hyperspectral techniques have shown improved signal specificity by retrieving the multiple chromophore concentrations.^100^^,^^106^^,^^210^^,^^211^ None of these technical innovations to improve the signal specificity have yet been adopted into commercial clinical products.

More anecdotally, PpIX fluorescence has been applied in oral cancer to detect squamous cell carcinoma and precancerous lesions that are difficult to identify, with topical 5-ALA giving selective accumulation in malignant cells and improving detection sensitivity.^212^^–^^214^ In lung cancer, 5-ALA (administered through inhalation or systemically) enables fluorescence bronchoscopy to improve detection of early lesions and dysplasia over white-light imaging alone.^55^ Similarly, systemic 5-ALA helps visualize abnormal areas in HPV-related cervical and vulvar neoplasia, enhancing lesion identification.^215^ High-grade squamous intraepithelial lesions of the cervix have been detected with fluorescence imaging and treated with PDT, in the combined APL-1702, a 5% hexyl-ALA HCl ointment.^216^ Urethral condyloma acuminatum has also been treated with ALA-based PDT.^217^ Breast cancer imaging with 5-ALA-PpIX fluorescence has also been reported,^218^ with multicenter clinical trials during surgical resection in progress.^218^ Beyond cancer, 5-ALA-PpIX fluorescence can assist in diagnosing bacterial and fungal infections, supporting more precise localization and fluorescence-guided debridement.^6^^,^^219^^–^^221^ Each of these areas has had considerable attention and shows promise, but further development and clinical testing are ongoing.

## 6 Biological Specificity—Metabolic Enzyme Activity, Inflammation, Invasion, and Infection

**Fig. 4:**
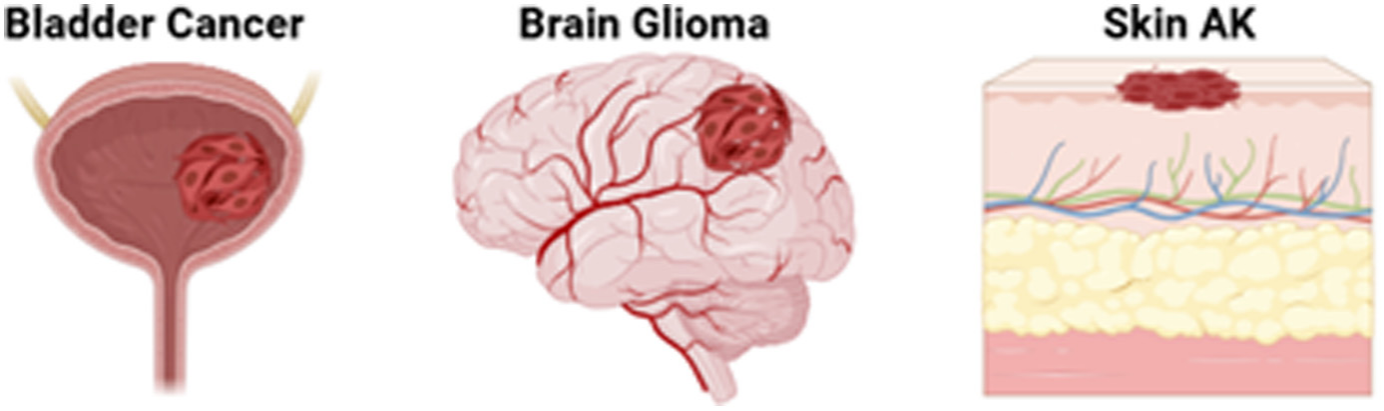
Three widely adopted clinical uses of 5-ALA-PpIX, namely, bladder cancer surgical visualization, glioma resection guidance, and skin lesion PDT, each having low uptake of 5-ALA, and hence, PpIX concentration, in the surrounding normal tissues relative to the target abnormal tissue.

**Fig. 5:**
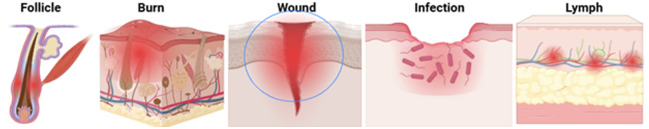
Examples of physical partitioning or localization of PpIX observed by fluorescence: in bacteria present in hair follicles; within burned or wounded tissues; at sites of bacterial infection; and in the lymph fluid and nodes associated with disease or tissue damage. Notably, the administration method (topical, systemic, etc.) can vary this localization pattern.

The application route of 5-ALA (topical, intracavity, oral, or intravenous) can affect the production and distribution of PpIX, with dynamic organ compartmentalization and clearance. Most successful clinical applications to date involve uses where the inherent background tissue has a very low PpIX concentration. The penetrance of 5-ALA in tissue is not generally thought to be a limitation due to the small size of the molecule^222^ so that it is the heme synthesis enzymes and their activity levels that are inherently low in the tissues such as muscle, brain, and bladder epithelium (Fig. 4). Nevertheless, inherent barriers to 5-ALA penetration could still be a factor because, for example, in brain, there is the blood-brain barrier,^223^ bladder has the urothelial lining,^224^ and, in normal skin, there is the keratinized epithelial layer^225^^,^^226^ (Fig. 5). Disruption of these barriers by tumor or inflammation then allows a combination of 5-ALA penetration and increased enzyme activity for PpIX synthesis for preferential site accumulation. In addition, there are cellular-level variations in PpIX production, which have been observed, leading to potentially high heterogeneity.

A related phenomenon is the observation of higher endogenous porphyrins in abnormal tissues or areas of high inflammation or bacterial infection in the absence of 5-ALA administration. For example, hair follicles have bacterial deposits that have red-fluorescence porphyrins, and amplification of PpIX by 5-ALA was demonstrated early in the use of PDT.^227^ This is true also in areas of infection,^228^^–^^230^ skin wounds,^230^ and burn tissue.^54^ Recently, PpIX has been observed in lymph fluid and nodes, as well as regions of normal tissue damage.^231^ These miscellaneous observations suggest producers or carriers of PpIX in bacteria and/or in immune cells such as macrophages.^232^ The localization of where the porphyrin is present should be further investigated to understand if this is a cause or effect within these carrier cells. For example, are the immune cells collecting the free PpIX from the fluid and plasma and subsequently locating to the wound areas or are they actively creating the PpIX and are simply locating within the wound areas?

## 7 Oxygen Sensing with PpIX

The observation that PpIX has an oxygen-dependent delayed fluorescence has been known since the mid 2000s, when Mik et al. first described this and others further elucidated the mechanisms.^39^^,^^40^ The delayed emission comes from energy transfer from reverse intersystem crossing from the populated PpIX triplet state.^41^^,^^233^ This excited state has a high likelihood for quenching by oxygen (quantum-mechanically permitted triplet-triplet transfer) so that, when the oxygen concentration is low, the reverse intersystem crossing yield increases and fluorescence emission occurs with a lifetime ∼3  ms. Even though this delayed fluorescence is orders of magnitude lower in intensity than the PpIX prompt fluorescence, its long lifetime allows the signal to be retrieved. This has been exploited to measure intracellular oxygen both *in vitro* and *in vivo*, for organ metabolism measurements^234^ and in human critical-care monitoring.^235^^–^^237^

**Fig. 6:**
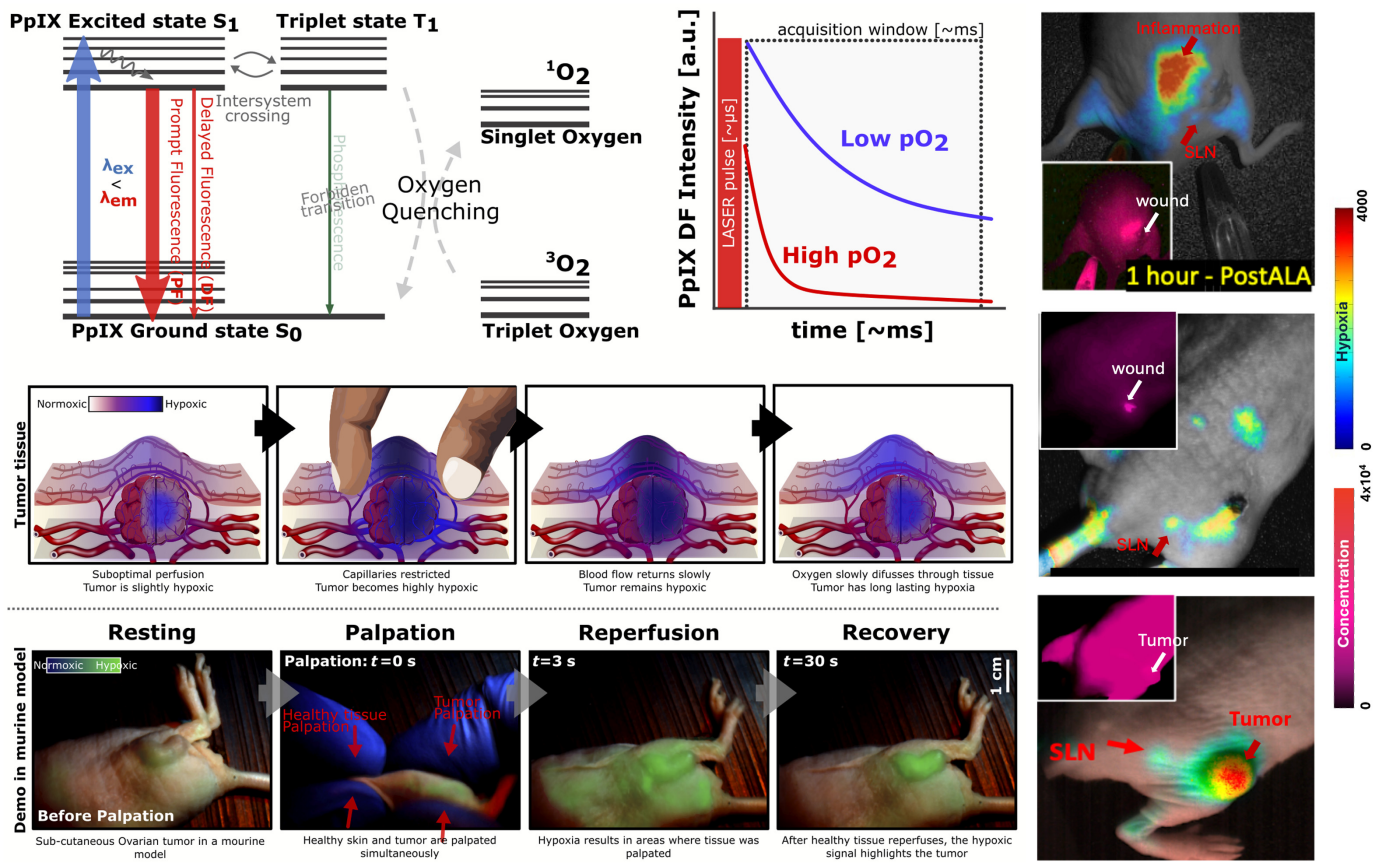
Depiction of hypoxia sensing with delayed PpIX fluorescence from reverse intersystem crossing and examples of hypoxia detected in tumors, sentinel lymph nodes (SLN), and regions of tissue inflammation. Both prompt and delayed fluorescence, representative of hypoxia, are shown. Tumor hypoxia through the PRESTO effect is displayed before and after pressing the tumor tissue and hence occluding the blood supply.^231^^,^^242^

Ongoing research includes the use of time-gated cameras that isolate the long-lived fluorescence in the time domain and imaging tissue hypoxia in real time.^238^ In addition, an increase in signal output has been reported through temporal oversampling of the signal, which improves hypoxia contrast.^239^ This is of significant interest because solid tumors are often hypoxic.^240^^,^^241^ A dual time-gate approach, where both delayed and prompt fluorescence are collected, has been employed to correct for PpIX concentration changes in the hypoxia image. Pressure applied to tissue induces transient hypoxia, with pressure-enhanced sensing of tissue oxygen (PRESTO) being demonstrated as a potential method to visualize differences in capillary refill of cancer *versus* normal tissues, illustrated in Fig. 6.^242^ Further studies have shown that hypoxia is present in lymph nodes and vessels and is also markedly present where there is local tissue damage, infection, or cancer.^231^ An important observation is the relationship between macrophages and myeloid cells as the potential origin of PpIX signals in high-grade gliomas.^232^ Together with the presence of PpIX in lymph nodes, inflammation, and/or damaged tissue (wounds, burns), this connects PpIX to the immune response. Further studies are needed to elucidate the mechanisms of PpIX-related immune response. These new phenomena may have a wide range of applications in medicine and biomedical science, given the serious limitations to imaging and understanding lymph and immune flow around the body.

## 8 Sub-Threshold Dose PDT Effects

**Fig. 7:**
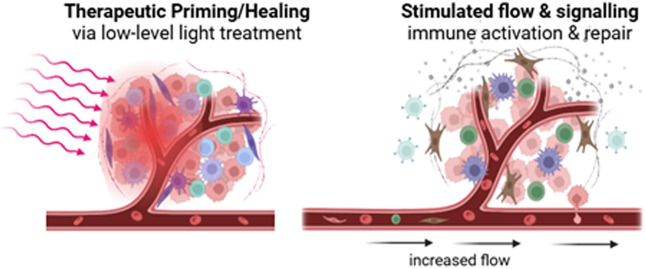
Illustration of the various effects that can occur in 5-ALA-PpIX PDT even at sub-threshold doses.

Photodynamic priming (PDP) describes PDT using light doses well below the threshold for necrosis, as illustrated in Fig. 7.^243^^–^^246^ Associated biological, biochemical, and biophysical effects are known to occur at low PDT doses, from using either a low 5-ALA dose, short incubation time, or lower light fluence. There is a broad class of sub-therapeutic changes that can occur as PDP alters the tumor microenvironment, and these may extend the impact of PDT beyond the standard above-threshold uses.^44^^,^^45^^,^^247^^–^^251^ Known mechanisms include upregulation of cytokines, along with sensitization of receptors and transient increases in permeability in the vessels and stroma that can enhance infiltration of biomolecules and immune cells and can be exploited for enhanced drug delivery. Examples of studies to date include combining PDP with radiation therapy or chemotherapy.^250^ PDP-induced anti-tumor immune stimulation has also been reported and may contribute to the observations of enhanced damage from fractionated light delivery.^50^^,^^51^^,^^53^^,^^252^

**Fig. 8:**
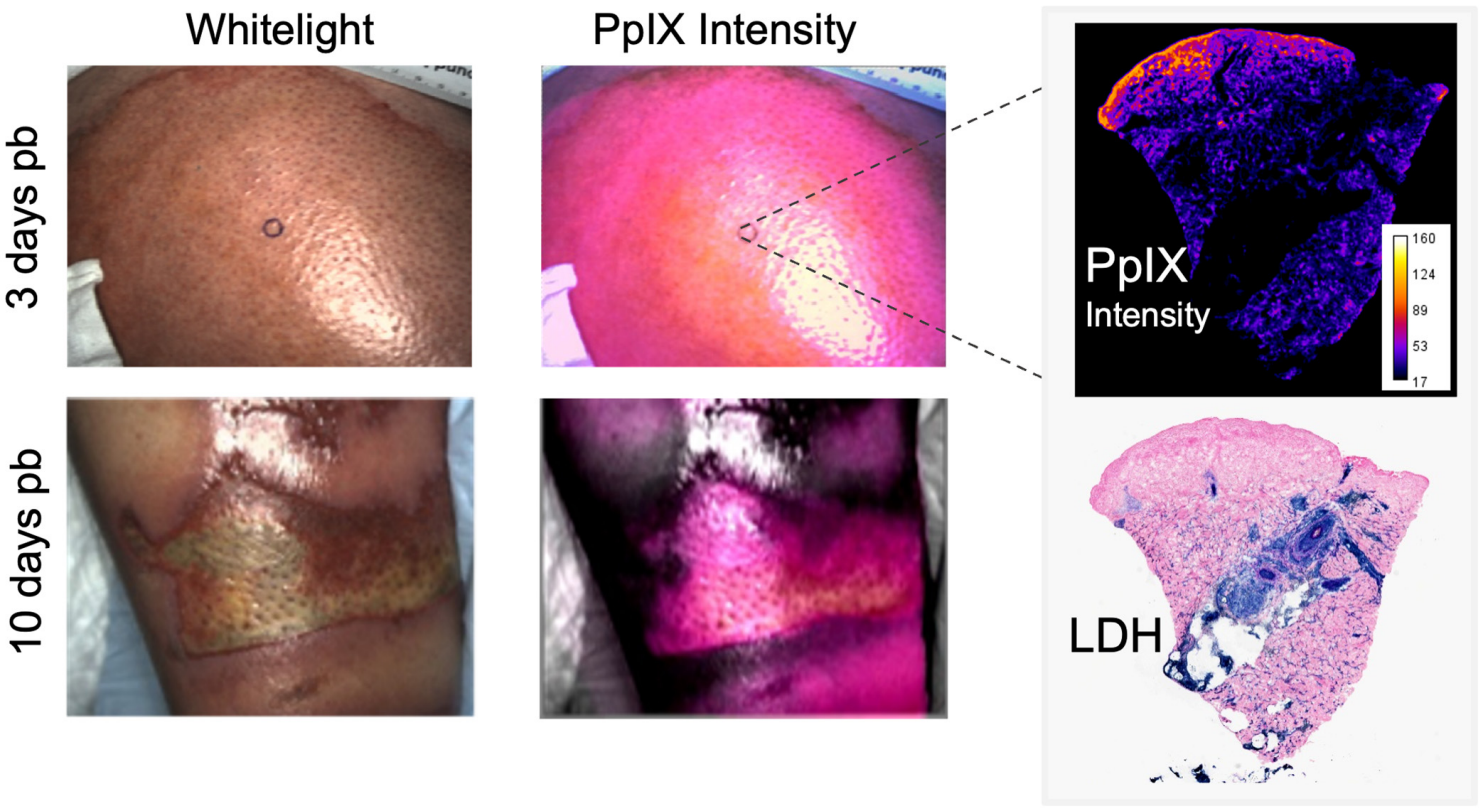
Example of PpIX intensity detected in human burns at 3 and 10 days post burn (pb). White light images are provided for visual reference. Biopsy area is indicated on 3 days pb subject. Biopsy was imaged through PpIX microscopy and is displayed together with LDH staining (blue), showing the reduced tissue viability in the upper layers where PpIX is present.

Burn injury treatment is an interesting potential application because of the known presence of PpIX in wounded tissues. This can generate low concentrations of ROS upon low-level light irradiation, enhancing wound healing by promoting cell proliferation, migration, and differentiation, as shown in recent *in vitro*, *ex vivo*, and *in vivo* animal studies.^253^^–^^259^ Low-dose 5-ALA-PpIX PDT has shown significant efficacy in enhancing burn-wound healing. In rodent models, it may promote burn-wound healing by activating stem cells in hair follicles,^254^ by modulating inflammation and by promoting angiogenesis, fibroblast proliferation, and collagen formation.^260^ Recent PpIX imaging findings in a mouse model and in human burns show burn tissue-specific accumulation of endogenous PpIX without 5-ALA administration, with some human data shown in Fig. 8.^54^ Endogenous PpIX localizes microscopically in the necrotic tissue and hair follicles. Macroscopic burn localization has also been recently observed for controlled porcine burns,^54^ but its correlation to burn depth has to be validated. Hence, additional studies in porcine models and humans are needed to fully unveil the molecular mechanisms of endogenous porphyrin accumulation in burns and its potential selectivity to necrotic tissue. Additional work highlights the potential of the marker as a theranostic agent for the treatment of burns.^261^ However, this principle still needs to be confirmed which type of porphyrin is present and studied in appropriate animal models of burns and, ultimately, in humans.

## 9 Alternate Photodynamic Excitation Modes

Metronomic PDT, in which both oral 5-ALA and fiberoptic interstitial light are delivered at very low rates, either continuously or in multiple small fractions over extended periods (hours-days-weeks),^262^^,^^263^ has been investigated in preclinical models.^202^^–^^205^ This approach parallels the paradigm of metronomic chemotherapy, with the philosophy of providing continuous activation of PpIX as it is produced. In orthotopic glioma tumor models, there was complete sparing of normal brain (neither necrosis nor apoptosis) while inducing significant apoptosis (also without necrosis) in glioma cells.

RadioDynamic therapy (RDT) or X-ray induced PDT (xPDT) is an emerging approach^96^ in which there is a wide range of options, from the use of radioactive nanoparticles to high-energy external X-ray beams. With 5-ALA-PpIX as the sensitizer, external beam irradiation has potential in glioma treatments. As discussed above, 5-ALA-PpIX fluorescence is already in common clinical use in guiding glioma surgery,^96^^,^^264^ and therapeutic X-rays have been combined with 5-ALA in rodent prostate tumors and brain tumors and show compelling evidence of synergistic cytotoxicity,^265^^–^^268^ although the mechanism of action is widely debated. These observations have been translated into ongoing clinical trials, facilitated by both components being standard-of-care in glioblastoma multiforme treatment. Improved outcomes are not yet established. There are several potential mechanisms of action, both direct and indirect, in this approach, including photosensitizer activation by secondary electrons and activation mediated by the Cherenkov light generated in tissue by the high-energy (MeV) X-rays.^269^^–^^272^ One mechanistic study showed that repeated application of 5-ALA in mice induced increased intracellular reactive oxygen species (ROS) that existed long after the ionizing irradiation.^273^^,^^274^ It is postulated that this is due to PpIX synthesis altering the generation and clearance of intracellular ROS. Alternatively, it is possible that the ionizing radiation may enhance intracellular PpIX accumulation by modulating transport pathways or upregulating enzymes involved in porphyrin biosynthesis, such as 5-ALAS and ferrochelatase activity alteration. Conversely, PpIX build up in cells with 5-ALA administration alone (without activation) is known to sensitize tumor cells to subsequent oxidative stress such as from ionizing radiation by influencing cellular redox state, mitochondrial function, and/or DNA repair pathways. In addition, both radiation and sub-therapeutic PpIX exposure independently generate ROS so that combining them may exceed the antioxidant buffering capacity of cancer cells, tipping the balance toward apoptosis or necrosis. 5-ALA-induced PpIX is produced in mitochondria, where even minimal ROS production could impair mitochondrial membrane potential and promote intrinsic apoptotic signaling. Moreover, both 5-ALA and radiation have been shown to induce immunogenic cell death, and their concurrent application may amplify antitumor immune responses through enhanced damage-associated molecular pattern release and antigen presentation.

Taken together, these multiple mechanisms support the rationale for ongoing clinical trials evaluating X-ray-induced PDT priming, such as in glioblastoma, where both 5-ALA and radiotherapy are elements of standard care.^96^^,^^264^

Sonodynamic therapy continues to be investigated, where high-intensity ultrasound irradiation is thought to activate the PpIX. There are several human trials ongoing to study the use of 5-ALA in glioma treatment.^275^^,^^276^ Preclinical studies have shown tumor eradication in experimental models.^277^ Both X-ray PDT and sonodynamic therapy have considerable practical advantages as they allow treatment of large and deep-seated tumor masses without the need for intraoperative, endoscopic, or interstitial fiberoptic light delivery, overcoming one of the main limitations of translating PDT into clinical practice.^278^ Clinical advancement has been supported by the commercial production of a new dedicated intravenous formulation of 5-ALA (SON5-ALA-001) for maximal penetration into the brain.

## 10 Limitations in 5-ALA-PpIX Use

The clinical applications of 5-ALA-PpIX have been limited by a few intrinsic factors, including the following.

• PpIX is highly photolabile compared with other fluorophores or photodynamic sensitizers. Its rapid photobleaching^157^^,^^279^^,^^280^ requires that long light exposure during fluorescence-guided surgery must be avoided, and for PDT, the loss of photoactivity can limit the delivery of an effective treatment dose. Conversely, the photobleaching can mean that 5-ALA-PpIX PDT is “self-limiting,” i.e., overdosing is avoided, thereby increasing the overall safety, and can be used as a dosimetry tool.^154^^,^^281^
• PpIX has active cellular efflux and diffusive-convective transport from the site of endogenous synthesis so that there is a trade-off between sensitivity and specificity in selecting the optimal time interval for fluorescence imaging or PDT treatment.^282^ Again, this can be considered a benefit for applications using systemic 5-ALA but can be a problem for locally applied (e.g., topical) 5-ALA.^17^^,^^283^
• The non-specificity of PpIX production is a complex issue in that, for example, inflammation or even simply high metabolic rate can generate significant PpIX levels in normal tissues, limiting some applications such as tumor-margin detection.^54^^,^^227^^,^^284^^,^^285^
• The maximum absorption of PpIX is with blue light, which limits imaging to the top mm or so of tissue.^286^^,^^287^ The alternative of red-light excitation (∼635  nm) gives deeper tissue sampling but comes at the expense of nearly 20-fold lower absorbance, resulting in a lower fluorescence signal and inability to measure the full spectrum. The deeper light penetration partially compensates for this by a larger sampling volume. For most PDT applications, the longest Q-band absorption peak around ∼635  nm is used to achieve adequate depth of treatment, but near-infrared photosensitizers can still give greater penetration.

Some of the more logistical factors that have limited use but can be solved through formulation, regulation, or engineering are:

• Autofluorescence with blue or red light is greater than with NIR fluorophores. This background significantly impacts the minimum detectable PpIX concentration, which is a technical challenge for some applications.
• Only oral and topical 5-ALA formulations are available currently, as FDA FDA-approved intravenous (IV) version has not been granted yet. Intraoperative applications (imaging or PDT) in particular would benefit from a systemic administration that avoids patients drinking fluids prior to general anesthesia. As mentioned above, commercial IV formulations are being evaluated in human trials for 5-ALA-PpIX SDT and PDT.
• PDT-induced pain has been a serious limitation in treating skin lesions^136^^,^^288^ and, for example, also led to trials for treating nail onychomycosis^289^ being discontinued. In dermatology, the pain has been partly mitigated by the simultaneous delivery of 5-ALA and light,^138^^,^^290^^,^^291^ or by the use of longer low fluence irradiations such as in daylight-PDT,^292^ with the trade-off of longer irradiation times that limit clinical use in high-volume settings.

## 11 Summary

**Fig. 9:**
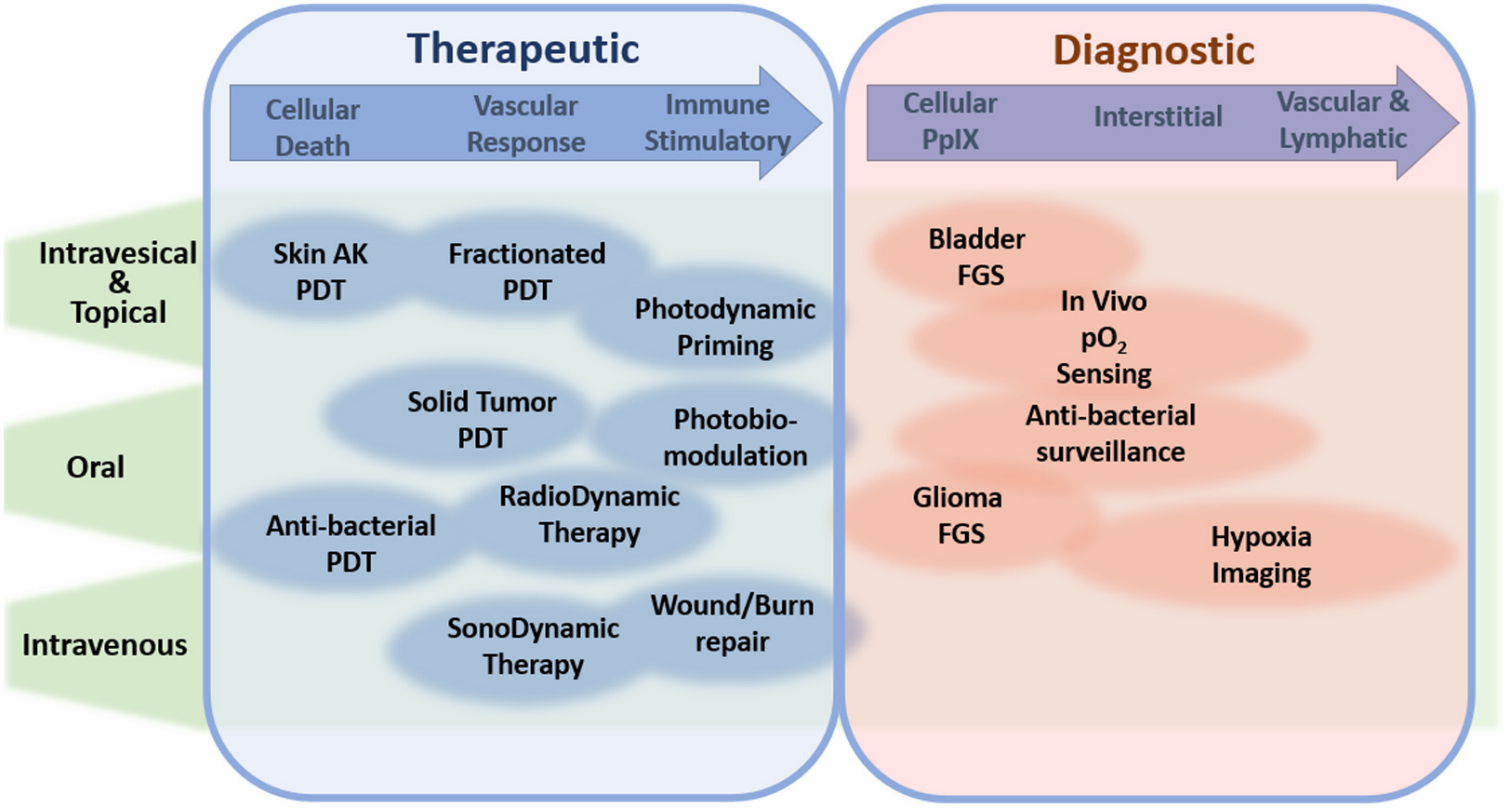
Illustration of the 5-ALA administration routes and therapeutic and diagnostic compartments affected or targeted for each application.

The range of applications in the use of 5-ALA-PpIX is large and growing well beyond its original indication as a PDT agent. These can be categorized as therapeutic or diagnostic applications, as outlined in Fig. 9. The administration route can vary from intravenous to oral to topical, and the individual application areas are roughly outlined in this figure, in terms of cellular-related, vascular-related, or immune-related, and each of these appears in both therapeutic and diagnostic areas.

Perhaps the strongest feature of the 5-ALA-PpIX system is the fact that the heme biosynthesis production pathway is a nearly ubiquitous cellular-driven metabolic cycle, which occurs in most tissue types at varying levels. This could be viewed as a weakness because PpIX may have a high normal tissue background, but its pervasive existence in biology provides a wide range of tissues in which it shows high contrast. 5-ALA-PpIX is also less molecularly specific than small molecule inhibitors, antagonists, or immune receptors that have site-specific binding for particular diseases. Nevertheless, it has the unique feature that it can be expressed across a broad range of organs and tissue types based on metabolism rather than the narrower definition of receptor expression within one disease type. This ability to be used across a broad range of diseases or tissue dysfunctions represents an “economy of scale” that can broaden its commercial uses over time. An analogy for this is fluorodeoxyglucose use in PET imaging, which has broad applicability across a range of pathologies, with the contrast being based upon the metabolic pathway of glucose uptake and respiration activity. Glucose uptake is ubiquitous across biology, but the unique contrast differences in diseased tissues are enough to make it useful, and its broad applicability across many cancers has value in the economy of scale. The 5-ALA-PpIX-Heme cycle has parallels to this, producing one of the few endogenous optically active agents, PpIX.

Another feature of the use of 5-ALA is that several formulations have been developed that are approved for human use for specific clinical indications but that share the common mode of action, through the heme synthesis cycle. Dermatology is the lead clinical specialty and continues to see expanded uses across more indications. Given the proven safety profile, it seems likely that additional applications will emerge from off-label uses. For example, although approved for AK treatment in the United States, it is used off-label in superficial basal cell and squamous cell carcinomas, infections (acne, leishmania, warts/condylomata), and skin rejuvenation, and several of these indications are already approved in other countries. Intravenous use of 5-ALA has not been adopted by a regulatory agency to date, but at least two commercial efforts are underway, and this would lead likely to wider use in surgical guidance.

Nearly all therapeutic strategies are thought to stem from singlet oxygen generation through triplet-state quenching by molecular oxygen, leading to cellular damage and death pathways. However, emerging modalities such as sonodynamic and radiodynamic therapy may involve additional mechanisms of action, whereas photodynamic priming may demonstrate therapeutic effects that are due not directly to cell kill but from sub-lethal changes in cell signaling or metabolism. These changes may prepare the tissue for adjuvant therapies or actively stimulate repair, such as in photobiostimulation or burn repair stimulation. The understanding and optimization of these latter activation modalities and use cases is still emerging, with high potential for wider use.

As a fluorescence guidance tool, the major applications to date utilize the prompt emission that reports on the PpIX concentration in tissue, whereas the less-developed applications exploit the delayed fluorescence signal due to low oxygen levels. Fluorescence image guidance in tumor surgery is the most widely used approach in the former, although there is growing interest in other indications. The delayed fluorescence has been developed as a commercial diagnostic tool already,^235^^,^^293^ and its use is likely to grow as the tools to sample it are invented and developed into usable devices. A hypoxia or oxygen-sensing agent that can image fast tissue dynamics from intracellular sites^242^^,^^294^^,^^295^ is an extremely unique feature of 5-ALA-PpIX in both surgical guidance and monitoring of tissue function.

Although the clinical use of 5-ALA-PpIX dates from the pioneering work of Kennedy, Pottier, and Pross in 1992 for treating skin cancer,^12^ there are still multiple emerging potential clinical applications arising from an expanded range of biophysical, biochemical, and biological characteristics or and from novel activation and detection/imaging technologies. There are also a growing number of companies involved in development and clinical trials in radiodynamic therapy, sonodynamic therapy, oxygen sensing, photostimulation, and photopriming. The range of diagnostic, therapeutic, and theranostic applications currently spans cancer, wounds, infection, and tissue repair and is likely to expand. Thus, in its fourth decade, the use of 5-ALA-PpIX in medicine remains an area of active research and development.

## Data Availability

Data sharing is not applicable to this article as no new data were created or analyzed.
